# Comparison of CT-like MRI sequences for preoperative planning of cochlear implantation using super-high-resolution CT as a reference

**DOI:** 10.1186/s41747-024-00538-x

**Published:** 2025-01-02

**Authors:** Ulysse Puel, Achille Beysang, Gabriella Hossu, Michael Eliezer, Bouchra Assabah, Khalid Ambarki, Pedro Augusto Gondim Teixeira, Alain Blum, Cécile Parietti-Winkler, Romain Gillet

**Affiliations:** 1 Guilloz Imaging Department, Central Hospital, University Hospital Center of Nancy, 29 Avenue du Marechal de Lattre de Tassigny, 54000 Nancy, France; 2 CIC, Innovation Technologique, Université de Lorraine, University Hospital Center of Nancy, Nancy, France; 3 University of Lorraine, INSERM, IADI, Nancy, France; 4 Department of Radiology, Centre Hospitalier National d’Ophtalmologie des Quinze-Vingts, 28 Rue de Charenton, 75012 Paris, France; 5 Faculty of Medicine and University Hospital, Department of Anatomy, University of Lorraine, 9 Avenue de la Forêt de Haye, 54500 Vandœuvre-lès-Nancy, France; 6 Siemens Healthcare SAS, Saint Denis, France; 7 Department of Otorhinolaryngology, Head and Neck Surgery, University Hospital, 29 avenue du Maréchal de Lattre de Tassigny, 54035 Nancy Cedex, France

**Keywords:** Cochlear implantation, Computed tomography, Chorda tympani nerve, Facial nerve, Magnetic resonance imaging

## Abstract

**Background:**

We evaluated the accuracy of magnetic resonance imaging (MRI) computed tomography (CT)-like sequences compared to normal-resolution CT (NR-CT) and super-high-resolution CT (SHR-CT) for planning of cochlear implantation.

**Methods:**

Six cadaveric temporal bone specimens were used. 3-T MRI scans were performed using radial volumetric interpolated breath-hold (STARVIBE), pointwise-encoding time reduction with radial acquisition (PETRA), and ultrashort time of echo (UTE) sequences. CT scans were performed on two scanners for SHR-CT and NR-CT acquisitions. Two radiologists evaluated accuracy based on preimplantation metrics and the ability to identify various anatomical structures, particularly the facial recess and round window. Wilcoxon rank-sum test and intraclass correlation coefficient (ICC) were used.

**Results:**

The facial nerve was always clearly visible (score ≥ 2) in the MRI, NR-CT, and SHR-CT scans (*p* ≥ 0.621). However, the chorda tympani nerve (CTN) was clearly visualized in UTE, STARVIBE, and PETRA sequences in only 33% (2/6 specimens, *p* = 0.016), 50% (3/6 specimens, *p* = 0.038), and 83% (5/6 specimens, *p* = 0.017) of cases, respectively, whereas it was always clearly visualized in SHR and NR-CT (*p* = 0.426). The round window (RW) was never visualized in MRI sequences (*p* ≤ 0.010), whereas it was identified in all cases in SHR and NR-CT (*p* = 1.000). There was a strong correlation between measurements obtained from MRI and CT modalities (ICC ≥ 0.837).

**Conclusion:**

MRI CT-like sequences assessed the facial nerve in all cases and the CTN in up to 87% of cases. However, the detection of the RW was insufficient for surgical planning. CT and MRI measurements were in agreement.

**Relevance statement:**

CT-like MRI sequences can image the anatomy of the facial recess and the length of the basal turn of the cochlea with similar accuracy as conventional CT, although they cannot image the round window.

**Key Points:**

- CT-like MRI sequences are not widely used in preoperative cochlear implantation imaging.
- CT-like sequences can image the facial recess as well as conventional CT.
- CT-like sequences can image the basal turn length of the cochlea as well as conventional CT.
- Round window depiction is not possible with CT-like MRI sequences.

**Graphical Abstract:**

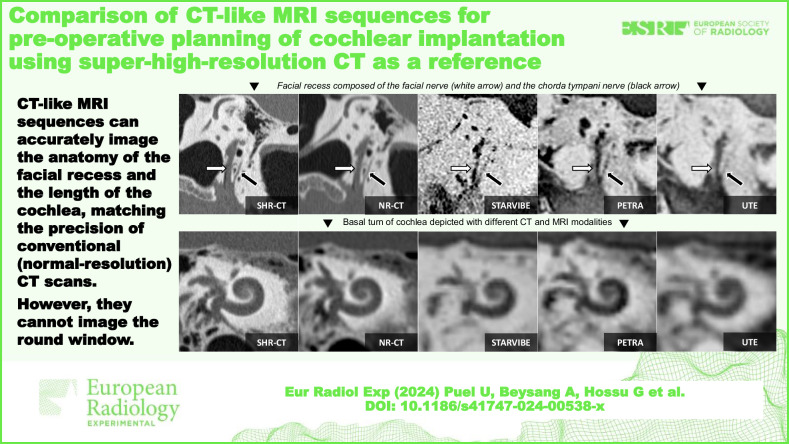

## Background

Investigation of anatomical structures for preoperative planning of posterior tympanotomy for cochlear implantation (CI) typically involves computed tomography (CT) and magnetic resonance imaging (MRI) [1]. There is a trend toward the primary use of MRI in CI evaluation, with advantages such as assessment of cochlear nerve caliber and cochlear patency. However, it is inaccurate in imaging bony landmarks. On the other hand, the preoperative evaluation of CI should identify critical and vulnerable structures such as the mastoid facial nerve (MFN) or the chorda tympani nerve (CTN) and anatomic variations that may complicate or contraindicate the procedure, usually identified by CT [2]. However, CI procedures are often performed in young children, where the use of CT results in radiation exposure and additional costs.

In this context, a volumetric spoiled gradient-echo “black bone” MRI sequence was recently used to depict the facial recess, bounded by the MFN and the CTN, with similar results to CT [3]. However, it did not address the other aspects of CI. In recent years, three-dimensional MRI sequences providing CT-like bone contrast have been developed for various musculoskeletal conditions with promising results but have not yet been investigated for temporal bone imaging [4].

The objective of this study was to compare the accuracy of various CT-like sequences for surgical planning of CI, using super-high-resolution CT (SHR-CT) as a reference.

## Methods

### *Ex vivo* specimens

Three head specimens obtained from the Department of Anatomy at the Lorraine’s University (Nancy, France) were frozen for preservation and then thawed for 48 h before CT and MRI in January 2024, resulting in six temporal bone specimens. All MRI and CT acquisitions were performed on specimens at a room temperature of 12 to 16 °C. Analysis was performed without knowledge of the clinical history of the subjects. Studies on anonymously donated specimens do not require institutional review board approval at our institution.

### CT and MRI protocols

Each specimen underwent conventional normal-resolution (NR)-CT and SHR-CT, as well as CT-like MRI sequences, namely radial volumetric interpolated breath-hold (STARVIBE), pointwise-encoding time reduction with radial acquisition (PETRA), and ultrashort time of echo (UTE), on the same day.

CT images were acquired using two different scanners: a SHR-CT scanner (Aquilion Precision, Canon Medical Systems, Ottawara, Japan) and an NR-CT scanner (Aquilion ONE, Canon Medical Systems, Ottawara, Japan). Both scanners used the same acquisition parameters: helical mode, 120 kVp, 220 mA, 1-s rotation time, and 7-cm field of view. Images were reconstructed using an FC80 reconstruction kernel with hybrid iterative reconstruction. The different CT acquisition and reconstruction parameters are summarized in Table 1. Of note, NR-CT used a 0.5-mm slice thickness with a 512^2^ matrix, while SHR-CT benefited from a 0.25-mm slice thickness and a 1,024^2^ matrix.

**Table 1:** Computed tomography technical parameters

	Super-high resolution	Normal resolution
Voltage (kVp)/current (mA)	120/220	120/220
Rotation time (s)	1	1
Detector	0.25 mm × 160 rows	0.5 mm × 320 rows
Voxel size (mm^3^)	0.25 × 0.25 × 0.25	0.5 × 0.5 × 0.5
Pitch factor (mm)	0.569	0.625
Field of view (cm)	7	7
Image matrix	1,024^2^	512^2^
Bedtime (s)	5.1	5.2
Dose length product (mGy*cm)	481.8	462.9

A 3-T MRI scanner (MAGNETOM Vida, Siemens Healthineers, Erlangen, Germany) with a 20-channel head coil was used, including axial PETRA, STARVIBE, and UTE sequences. The STARVIBE sequence is a T1-weighted three-dimensional sequence acquired radially [5]; the PETRA and UTE sequences are three-dimensional T2-weighted sequences with an ultrashort echo time [6]. MRI protocols are summarized in Table 2.

**Table 2:** Magnetic resonance imaging technical parameters

	STARVIBE	PETRA	UTE
Repetition time/echo time (ms)	500/246	330/8	460/4
Flip angle (°)	3	2	5
Spikes per segment	120	320	304
Voxel size (mm^3^)	0.6 × 0.6 × 0.6	0.6 × 0.6 × 0.6	0.6 × 0.6 × 0.6
Gap (mm)	0.6	0	0.12
Acquisition field of view	200 × 200	200 × 200	192 × 192
Resolution	320 × 320	320 × 320	304 × 304
Acquisition time (min:s)	6:48	5:07	2:28

### Image analysis

Two head and neck radiologists with three (A.B.) and four (U.P.) years of clinical experience independently analyzed the images. Before reading, a training session was conducted by a third radiologist (R.G.) with ten years of experience on 10 UHR-CT scanners not included in this study. The procedure was repeated with at least a 2-week interval between each session to assess intraobserver agreement.

#### Qualitative image analysis

**Fig. 1:**
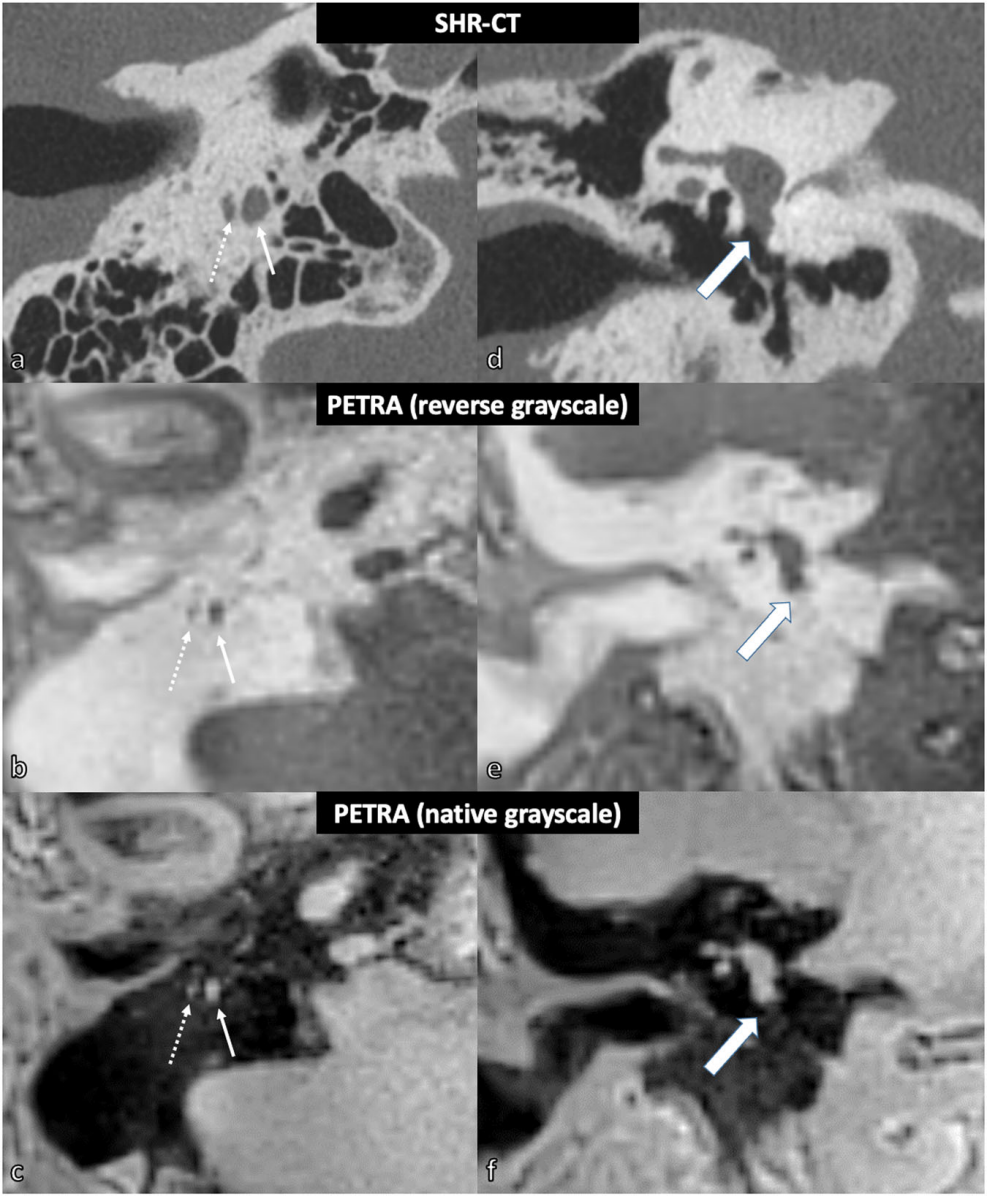
Right mastoid portion of the facial nerve (MFN), bony segment of the chorda tympani nerve (CTN), and round window niche with SHR-CT and PETRA MRI sequences (example in living patient). The mastoid portion of the facial nerve (MFN: solid white arrow) and the origin of the bony segment of the chorda tympani nerve (CTN: dotted white arrow) are clearly visible in SHR-CT (**a**) and PETRA MRI sequence with inverted grayscale (**b**) and native grayscale (**c**). The round window niche (large white arrow) is clearly visible in SHR-CT (**d**) but remains indistinguishable in the inverted (**e**) and native (**f**) grayscale PETRA MRI sequence. MRI, Magnetic resonance imaging; SHR-CT, Super-high-resolution computed tomography

**Fig. 2:**
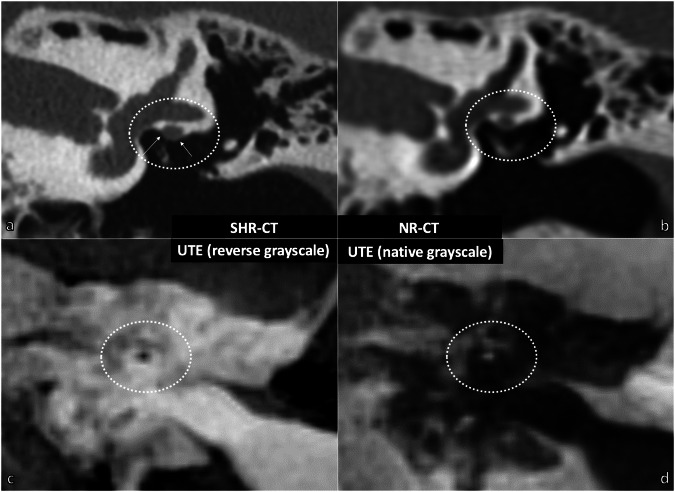
Osseous coverage of the tympanic portion of the facial nerve analyzed with CT and UTE-MRI sequences in the coronal plane (example in living patient). **a** SHR-CT and (**b**) NR-CT showing a clearly visible osseous coverage (white arrows), whereas it is indistinguishable in the UTE-MRI sequence with inverted grayscale (**c**) and native grayscale (**d**), as well as the air-bone interface in the dotted circle. CT*,* Computed tomography; MRI, Magnetic resonance imaging; NR*,* Normal resolution; SHR*,* Super-high resolution; UTE, Ultrashort time of echo

**Fig. 3:**
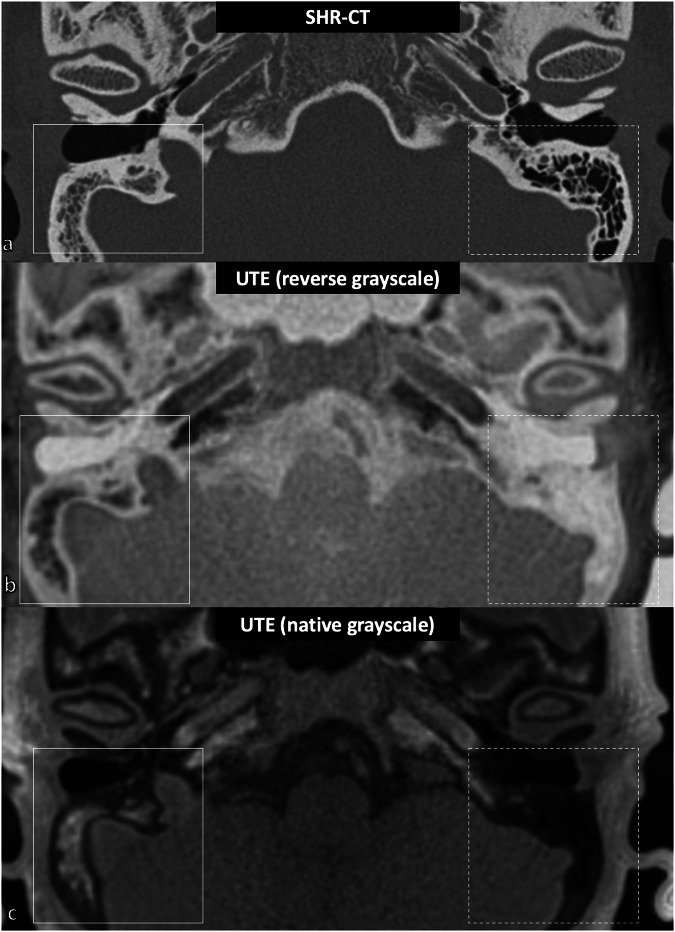
Pneumatization of mastoid air cells in SHR-CT and UTE-MRI (example in living patient) in the axial plane. **a** Mastoid air cells in SHR-CT. Note the hypopneumatization of the right mastoid air cells (white box), whose bony appearance is comparable to the zygomatic process of the temporal bone, and the standard pneumatization of the left mastoid air cells (dotted box), with their corresponding aspects in the UTE-MRI sequence with reverse grayscale (**b**) and native grayscale (**c**). CT, Computed tomography; MRI, Magnetic resonance imaging; NR, Normal resolution; SHR, Super-high resolution; UTE, Ultrashort time of echo

Three elements were selected based on the perceived difficulty of visualization on NR-CT according to previous studies, namely the mastoid portion of the facial nerve (MFN), the bony segment of CTN, and the round window (RW) [3, 7, 8]. A 4-point visibility scale was used: 0 = not demonstrated; 1 = seen only in a minority of sections, or uncertain visualization relative to the background; 2 = seen in the majority of sections with uncertain visualization relative to the background in a minority of sections; 3 = seen in continuity on all sections [3]. A visibility score of 2 or higher corresponded to a clear visualization (Fig. 1). The osseous coverage of the tympanic portion of the facial nerve was quoted using a qualitative score (0 = no coverage; 1 = questionable coverage; 2 = normal coverage) (Fig. 2). The mastoid air cells’ pneumatization and opacification (*i.e*., fluid filling) were rated as follows: 0 = no pneumatization/opacification; 1 = hypopneumatization/partial opacification; 2 = normal pneumatization/complete opacification (Fig. 3). To identify a potential associated diagnosis (*i.e*., a third window syndrome), the bony coverage of the superior semicircular canal (SSCC) was quoted using a qualitative score (0 = no coverage; 1 = questionable coverage; 2 = normal coverage) in the Pöschl plan [9].

#### Quantitative image analysis

**Fig. 4:**
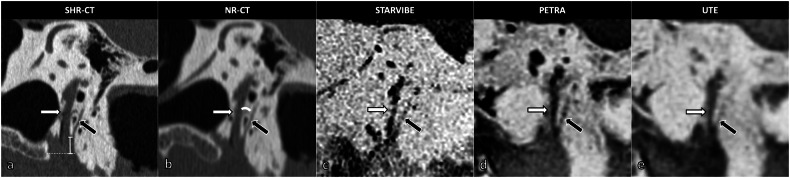
Right facial recess imaged with different CT and MRI modalities in the parasagittal plane (anatomic specimen). **a** SHR-CT showing the distance between the origin of the chorda tympani nerve (black arrow) and the stylomastoid foramen measurement method. **b** NR-CT showing the chorda-facial angle measurement method between the mastoid portion of the facial nerve (MFN: white arrow) and the origin of the bony segment of the chorda tympani nerve (CTN: black arrow). Facial recess composed by MFN and the bony segment of CTN imaged in STARVIBE (**c**), PETRA (**d**), and UTE (**e**) MRI sequences. CT, Computed tomography; MRI, Magnetic resonance imaging; NR, Normal resolution; SHR, Super-high resolution; UTE, Ultrashort time of echo
- The angle between the origin of the CTN and the MFN in an oblique para-coronal plane (Fig. 4b and Supplementary Fig. S1b) [1].
- The minimum thickness of bone separating the SSCC from the middle cranial fossa in the Pöschl plane (Fig. 5a and Supplementary Fig. S2a) [9].

The two readers measured the following parameters:

- The craniocaudal distance between the origin of the CTN and the stylomastoid foramen (CTN-SMF distance) in a parasagittal plane (Fig. 4a and Supplementary Fig. S1a) [1].

**Fig. 5:**
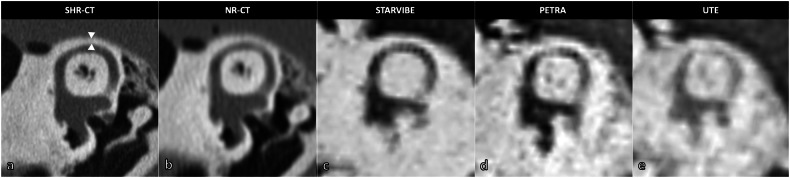
Bony coverage of the superior semicircular canal using different CT and MRI modalities in the Pöschl plane (anatomic specimen). **a** SHR-CT showing the measurement method (distance between the two arrowheads). **b** Pöschl plane in NR-CT. Pöschl plan acquired in STARVIBE (**c**), PETRA (**d**), and UTE (**e**) MRI sequences. CT, Computed tomography; MRI, Magnetic resonance imaging; NR, Normal resolution; SHR, Super-high resolution; UTE, Ultrashort time of echo
- The length of the basal turn of the cochlea calculated for implant selection, as the distance from the center of the RW to the farthest point on the opposite wall of the cochlea, passing through the modiolus in a double oblique reformatted plane (Fig. 6a) [10].

**Fig. 6:**
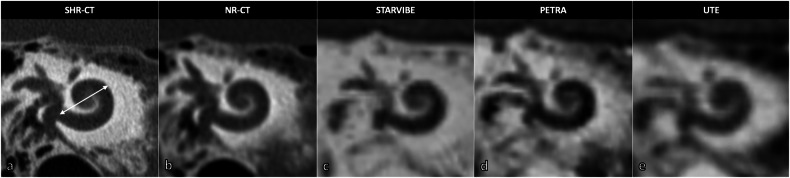
Basal turn of cochlea imaged with different CT and MRI modalities (anatomic specimen). **a** Basal cochlear turn SHR-CT reformation showing the measurement of the basal turn length of the cochlea as the greatest distance from the round window, through the modiolar axis, to the lateral wall (double arrow). **b** Basal cochlear turn NR-CT reformation. Basal turn of cochlea imaged in STARVIBE (**c**), PETRA (**d**), and UTE (**e**) MRI sequences. CT, Computed tomography; MRI, Magnetic resonance imaging; NR, Normal resolution; SHR, Super-high resolution; UTE, Ultrashort time of echo
- The angle between the tegmen tympani superiorly and the sigmoid sinus inferiorly in the sagittal plane as the sinodural angle (Supplementary Fig. S3a).

### Statistical analysis

Descriptive statistics of ordinal qualitative variables were expressed as medians with an interquartile range (Table 3). Given the non-normal distribution confirmed by the Shapiro–Wilk test, Wilcoxon rank-sum tests with Bonferroni correction were employed to identify differences between CT-like MRI sequences, NR-CT, and SHR-CT. Cohen *κ* statistics with linear weighted was used to measure interobserver and intraobserver agreement. A *κ* value in the range of 0 to 0.20 was interpreted as slight agreement, 0.21 to 0.40 as fair, 0.41 to 0.60 as moderate, 0.61 to 0.80 as substantial, and 0.81 to 1 as almost perfect [11].

**Table 3:** Qualitative measurement for each reader in computed tomography and magnetic resonance imaging

Qualitative measurement		Computed tomography		Magnetic resonance imaging			*κ* intraobserver	*κ* interobserver
		SHR	NR	STARVIBE	PETRA	UTE		
Mastoid segment of facial nerve	R1	3 (3; 3)	3 (3; 3)	3 (3; 3)	3 (3; 3)	3 (3; 3)	1.000***	0.308***
	R2	3 (3; 3)	3 (3; 3)	3 (3; 3)	3 (2.75; 3)	3 (3; 3)	0.806***	
Osseous coverage of the facial nerve	R1	3 (3; 3)	2 (2; 2)	0 (0; 0)	0 (0; 0)	0 (0; 0)	1.000***	0.870***
	R2	3 (3; 3)	1.5 (1; 2)	0 (0; 0)	0 (0; 0)	0 (0; 0)	0.963***	
Bony segment of chorda tympani	R1	3 (3; 3)	3 (2.75; 3)	1.5 (1; 3)	2 (2; 2)	1 (1; 2)	0.843***	0.834***
	R2	3 (3; 3)	3 (2; 3)	1.5 (1; 3)	1 (1; 2)	1 (1; 1)	0.980***	
Round window	R1	3 (3; 3)	3 (3; 3)	0 (0; 0)	0 (0; 0)	0 (0; 0)	0.919***	0.903***
	R2	3 (3; 3)	3 (3; 3)	0 (0; 0)	0 (0; 0)	0 (0; 0)	1.000***	
Superior semicircular canal bony cover	R1	2 (2; 2)	2 (2; 2)	2 (2; 2)	2 (1; 2)	2 (1; 2)	0.556***	0.333***
	R2	2 (2; 2)	2 (2; 2)	2 (2; 2)	2 (2; 2)	2 (1; 2)	0.876***	
Mastoid cells pneumatization	R1	1.5 (1; 2)	1.5 (1; 1.5)	1 (0.75; 2)	1.5 (1; 2)	1.5 (1; 2)	0.618***	0.477***
	R2	2 (1; 2)	1 (1; 2)	1 (0; 2)	1 (1; 2)	1 (1; 2)	0.883***	
Mastoid cells opacification	R1	1 (0; 1)	1 (0; 1)	1 (0; 1)	1 (0; 1)	1 (0; 1)	0.667***	0.619***
	R2	0.5 (0; 0.5)	0.5 (0; 0.5)	0.5 (0; 0.5)	0.5 (0; 0.5)	0.5 (0; 0.5)	0.905***	

Data are reported as median, and numbers in brackets are interquartile ranges; significant values obtained by Wilcoxon ranked sum test with Bonferroni correction are indicated as follows: ***p < 0.001. *NR* Normal resolution, *R* Reader, *SHR* Super-high resolution

Descriptive statistics for quantitative variables were reported as mean ± standard deviation (Table 4). The intraclass correlation coefficient (ICC) with two-way random effects was used to assess the agreement between CT-like MRI sequences and NR-CT in comparison to SHR-CT and to measure interobserver and intraobserver agreements. Values < 0.50, ≥ 0.50 but < 0.75, ≥ 0.75 but < 0.90, and ≥ 0.90 were indicative of poor, moderate, good, and excellent reliability, respectively [11].

**Table 4:** Quantitative measurement for each reader in computed tomography and magnetic resonance imaging

Quantitative measurement		Computed tomography		Magnetic resonance imaging				
		SHR	NR	STARVIBE	PETRA	UTE	ICC intraobserver	ICC interobserver
ChT-SMF distance (mm)	R1	0.8 ± 1.5	0.8 ± 1.6	1.0 ± 1.7	0.9 ± 1.6	NA	0.980	0.940
	R2	1.0 ± 1.5	1.0 ± 1.6	1.3 ± 1.4	1.1 ± 1.6	NA	0.997	
	ICC	Reference	1.000	0.994	1.000	0.984		
ChT-mFN angle (°)	R1	27.3 ± 8.1	27.3 ± 8.3	28.4 ± 8.5	29.3 ± 8.5	NA	0.925	0.924
	R2	26.0 ± 8.3	26.0 ± 9.4	29.1 ± 9.3	32.0 ± 10.9	NA	0.983	
	ICC	Reference	0.986	0.963	0.952	0.907		
SSCC bony cover thickness (mm)	R1	1.0 ± 1.0	1.1 ± 0.9	1.2 ± 0.9	1.62 ± 0.7	1.1 ± 0.8	0.907	0.728
	R2	0.7 ± 0.6	0.7 ± 0.6	0.9 ± 0.5	1.2 ± 0.6	0.8 ± 0.6	0.976	
	ICC	Reference	0.992	0.980	0.853	0.945		
Basal turn of the cochlea length (mm)	R1	8.8 ± 0.9	8.8 ± 0.8	8.8 ± 0.9	8.8 ± 0.9	8.9 ± 0.9	0.968	0.798
	R2	8.5 ± 0.6	8.7 ± 0.5	8.7 ± 0.6	8.7 ± 0.9	9.1 ± 0.9	0.979	
	ICC	Reference	0.998	0.981	0.982	0.979		
Sinodural angle (°)	R1	51.5 ± 4.6	52.1 ± 4.9	49.4 ± 2.7	53.7 ± 4.6	52.3 ± 4.2	0.823	0.737
	R2	53.4 ± 4.4	54.8 ± 5.5	51.9 ± 3.8	53.0 ± 6.6	53.1 ± 6.4	0.950	
	ICC	Reference	0.930	0.880	0.837	0.889		

Data are reported as mean ± standard deviation

*ChT-mFN angle* Angle between the origin of the bony segment of the chorda tympani nerve and the mastoid portion of the facial nerve, *ChT-SMF distance* distance between the chorda tympani nerve and the stylomastoid foramen, *ICC* Intraclass correlation coefficient, *NR* Normal resolution, *SHR* Super-high resolution, *SSCC* Superior semicircular canal

Statistical significance was set at *p* < 0.050. Statistical analysis was performed using RStudio (version 2022.07.2) and Jamovi software (version 2.3.18.0).

## Results

### Qualitative results

All qualitative scores are summarized in Table 3 along with their inter- and intraobserver agreement, all of which were excellent (*p* < 0.001).

The MFN was always clearly delineated with SHR-CT and NR-CT as well as in the CT-like MRI sequences. No significant differences in the scores were observed between the CT-like MRI sequences and the SHR acquisition (*p* ≥ 0.621).

The bony segment of the CTN was clearly visible in 83% of cases with NR-CT, in 100% with SHR-CT, and in 83% of cases with the PETRA sequence. However, it was clearly visualized in only 50% and 33% of cases with the STARVIBE and UTE sequences, respectively. This difference in visibility scores between each CT-like MRI sequence and the SHR acquisition was statistically significant (*p* ≤ 0.038). It was not depicted in one case, with the UTE sequence only. All the remaining cases showed a visualization score of 1.

The RW was consistently indistinct in the CT-like sequences, whereas it was clearly visible in all NR-CT and SHR-CT acquisitions (*p* ≤ 0.004). Thus, measurements of its niche were impossible for all CT-like MRI sequences. Also, the facial bony coverage in the middle ear cavities was not visible in the CT-like sequences, whereas it was clearly visible in different CT acquisitions (*p* < 0.001).

There was no evidence of SSCC dehiscence (score = 2) in SHR-CT, NR-CT, and STARVIBE images, so the bony coverage was always well visualized. However, uncertainty (score = 1) was observed in 16% of UTE and 33% of PETRA sequences. However, this difference did not reach statistical significance (*p* ≥ 0.308).

Four of the 6 mastoid air cells were normally pneumatized (score = 2), while 2 were hypopneumatized (score = 1) with SHR-CT and NR-CT. All mastoid air cells were partially opacified. For both readers, there was no significant difference between all pneumatization and opacification ratings of CT-like MRI sequences, NR-CT, and SHR-CT (*p* < 0.017 and *p* < 0.001 for pneumatization and opacification, respectively).

### Quantitative results

All quantitative values are summarized in Table 4, along with their interobserver and intraobserver agreement. Global interobserver agreement was excellent (ICC = 0.986), and global intraobserver agreement was also excellent (ICC = 0.991 and ICC = 0.997 for reader 1 and reader 2, respectively). In detail, all were good to excellent (ICC ≥ 0.798) except for the interobserver agreement of SSCC bone coverage thickness and sinodural angle, which was moderate (ICC = 0.728 and ICC = 0.737, respectively).

The distance between the CTN and the SMF, as well as the angle between the origin of the CTN and the MFN, showed a good to excellent agreement with SHR-CT and NR-CT in all CT-like MRI sequences (ICC ≥ 0.907).

Both STARVIBE and UTE sequences showed almost perfect agreement with SHR-CT in measuring SSCC bone coverage (ICC ≥ 0.945). While the PETRA sequence showed strong agreement with the SHR-CT acquisition, it was slightly inferior, but the agreement remained robust (ICC = 0.853).

In addition, the basal turn length of the cochlea showed almost perfect agreement between all CT-like MRI sequences and SHR-CT acquisitions, especially remarkable for the PETRA sequence (ICC = 0.982).

Finally, the sinodural angle showed an almost perfect agreement between SHR-CT and NR-CT (ICC = 0.930), but a slight inferior agreement when comparing SHR-CT and all CT-like MRI sequences (ICC ≥ 0.837).

## Discussion

To our knowledge, this is the first study comparing different CT-like MRI sequences to NR-CT and SHR-CT acquisitions in temporal bone imaging. We demonstrated the ability of CT-like sequences to define temporal bone anatomy in preoperative planning for CI, especially for posterior tympanotomy and basal turn length of the cochlea measurement, but excluding the RW, regardless of the sequence. Almost all sequences showed the facial recess, with variable performance, but with excellent agreement with SHR-CT, and all sequences allowed accurate measurement of basal turn length of the cochlea.

Surgical approaches may need to be adapted based on anatomical variations or malformations of the temporal bone, which can increase the complexity of surgery and lead to longer intraoperative times and a higher risk of complications compared to patients with standard anatomy. Standard MRI images already provide much of the information relevant to the surgical anatomy of mastoid approaches, such as mastoid pneumatization, the position of the jugular bulb, and posterior wall of the external auditory canal [12]. The MFN and the bony segment of the CTN are two critical structures for preoperative surgical planning because they form the facial nerve recess through which the surgeon will pass during the mastoidectomy procedure, exposing them to iatrogenic injury [1, 3, 12]. However, they are not consistently visible on standard MRI sequences. In this setting, a volumetric spoiled gradient-echo “black bone” MRI sequence was previously used and allowed to demonstrate the MFN and CTN in 91% and 81%, respectively, compared to CT [3]. The “black bone” sequence is advantageous for visualizing neural structures, which appear as high signal intensity in contrast to adjacent bone and air, whereas they appeared as low signal intensity in the MRI CT-like sequences tested in our study. We found higher performance for the CT-like MRI sequences tested for the MFN, as it was always depicted in all sequences. This could be due to the fact that the “black bone” sequence was tested on a 1.5-T MRI scanner, whereas we performed our study on a 3-T MRI scanner, and related to the superior “bone” contrast provided by the tested sequences compared to the spoiled gradient-echo “black bone” sequence. In our study, the CTN was almost always visualized. However, visualization scores were variable and better with the PETRA sequence, which clearly depicted 83% of the CTN, giving it the same performance as NR-CT. With the STARVIBE and UTE sequences, the CTN was clearly visualized in only half and one-third of the cases, respectively, although it was not visualized in one unique UTE case. The PETRA sequence also showed better agreement in measurements with SHR-CT, except for SSCC bony coverage. The PETRA sequence outperformed the STARVIBE sequence due to its construction as a zero echo time sequence, which allows better visualization of osseous structures due to its ultrashort T2 relaxation time. In contrast, the T1-weighted STARVIBE sequence visualizes only the T1 signal of bone by contrast with adjacent structures [4]. The PETRA sequence also outperformed the UTE sequence, probably due to its higher acquisition and reconstruction matrices. The lack of signal of SSCC in some studies using MRI CT-like sequences may be related to the air-bone interface present at the roof of the SSCC. Another possible explanation may be related to the presence of hemorrhagic or mucinous deposits in SSCC from cadaveric specimens [13]. Furthermore, the presence or absence of a third window syndrome is not in itself a contraindication to CI. More precisely, there was no difference in postoperative efficacy between CI recipients with and without SSCC dehiscence [14], and another study even suggested that CI may be beneficial in such cases [15]. Therefore, we believe that CT-like MRI sequences can be used for surgical planning in CI because they showed the facial recess, especially with the PETRA sequence, with the same accuracy as NR-CT. Also, the measurement of the length of the basal turn of the cochlea had an excellent correlation with SHR-CT in all sequences. In addition, the relatively shorter acquisition time (5:07 min for PETRA *versus*. 6:43 min:s for “black bone”) argues for its incorporation into a standard MRI protocol.

The major drawback of these sequences, as for the “black bone” sequence, was their inability to visualize the RW, which is the site of penetration of the device into the inner ear for CI [3]. The PETRA sequence could not visualize the middle ear structures due to its insufficient spatial resolution and its low ossicular T2 signal contrast, while the STARVIBE sequence could not discriminate the signal from air and bone due to the lack of T1 signal. On the other hand, some studies suggest that MRI alone can effectively perform preoperative evaluation for CI in the absence of clinical evidence of cochlear dysplasia or malformation syndrome [16]. The RW morphology itself is not described in all preoperative CI checklist reports, and its accessibility is rather based on the combination of facial recess and cochlear position anatomical factors [12, 17]. Conversely, other pathological conditions require visualization of the RW niche, which predicts CI efficacy (*e.g*., cochlear dysplasia, labyrinthine ossification), CI difficulty, and risk of complications (*e.g*., detection of otospongiotic plaques, especially in the RW niche) [16, 18]. In this context, temporal bone CT has already been described as a very good test to exclude access difficulties to the RW (sensitivity 46%; specificity 92%) [17]. However, there is no consensus regarding the posterior tympanotomy approach.

Many authors consider the visibility of the RW to be a critical factor, as it can guide this procedure (*e.g*., cochleostomy *versus* RW approach) [18–20]. In general, MRI CT-like sequences may be performed in certain indications, such as simple cholesteatoma, without evidence of middle ear invasion on MRI. Alzahrani et al even suggested the use of MRI fusion with previous CT scans for follow-up [21]. In the author’s experience, a previous CT scan may be available in the patient’s medical record and should not be repeated, as RW morphology does not change over time. Finally, this approach could streamline the patient journey by eliminating the need for additional CT scans. However, it should be noted that the bony coverage of the tympanic segment of the facial nerve could not be imaged with CT-like MRI sequences, which may limit the utility of CT-like MRI in preoperative planning for middle ear approaches.

This study has several limitations. First, the study population was small and included only normal examinations. Therefore, larger studies, including pathological findings, are needed to confirm our results, especially regarding CI contraindications such as cochlear ossification. The spatial resolution of the CT-like MRI sequences remained low (*i.e*., 1.2 mm *versus* 0.5 mm for NR-CT). Only one manufacturer’s CT-like MRI sequences were tested. The UTE sequence was relatively short (approximately 2 min), so an optimized longer sequence may provide better results. We monitored the temperature during all MRI scans, which ranged from 12 to 16 °C. Despite our efforts to maintain a consistent temperature, there may be some variability among frozen cadaveric specimens, which could result in a slight reduction in T2 and a relative sparing of T1 relaxation times in the affected temperature ranges [22]. However, signal intensity should be preserved during this variation, and our results point in this direction. In addition, we did not consider other factors, such as postmortem delay before MRI. Therefore, we may expect better results from PETRA and UTE sequences in studies performed on living subjects and using a head coil with more channels (*i.e*., 64 instead of 20 channels) as in our figures shown (Figs. 1–3 and Supplementary Fig. S2). In this study, we used only the Pöschl plan to assess the bony coverage of the SSCC and did not use the Stenvers plan for confirmation. However, none of the cases in this sample were doubtful, which did not interfere with our analysis. *In vivo* analysis may also be associated with less or no opacification of the mastoid and middle ear cavities, whereas cadaveric specimens often have significant opacification. On the other hand, cadaveric specimens are free of motion artifacts, which is not the case *in vivo*. Digital volume tomography is another valuable CT technique that offers 0.125-mm slice thickness and high spatial resolution along with very low radiation dose values, approximately 1/100 to 1/300 that of conventional CT, but it could not be compared with CT-like MRI in this study [23]. It also allows postoperative evaluation of CI with less susceptibility to metallic artifacts than conventional CT, which is not possible with MRI. Finally, it is important to emphasize that we have not yet evaluated the technique in the pediatric population, where the benefits of avoiding ionizing radiation would be significant [24].

In conclusion, newer MRI CT-like sequences can assess facial recess anatomy and basal turn length of the cochlea with the same accuracy as NR-CT for CI preoperative planning. In particular, measurements derived from the PETRA sequence showed the highest correlation with the reference SHR-CT acquisition. However, their performance for RW imaging remains inadequate and should be improved to avoid the need for complementary CT.

## Supplementary information

**Additional file 1**: **Supplementary Fig. S1**. Right facial recess imaged with different CT and UTE-MRI sequences in the parasagittal plane (example in living patient). **a** SHR-CT showing the distance between the origin of the chorda tympani nerve (white dotted arrow) and the stylomastoid foramen measurement method. **b** NR-CT showing the chorda-facial angle measurement method between the mastoid portion of the facial nerve (MFN: white arrow) and the origin of the bony segment of the chorda tympani nerve (CTN: dotted white arrow). Facial recess composed of the MFN and the bony segment of the CTN in UTE sequences in inverted grayscale (**c**) and native grayscale (**d**). CT, computed tomography; MRI, magnetic resonance imaging; NR, normal resolution; SHR, super-high resolution; UTE, ultrashort time of echo. **Supplementary Fig. S2**. Superior semicircular canal and tympanic bony coverage in a 19-year-old patient followed for an operated cholesteatoma in NR-CT and STARVIBE MRI sequences (example in living patient). Superior semicircular canal bony coverage in the Pöschl plane in NR-CT (**a**) and PETRA reverse grayscale (**b**) and native grayscale (**c**) MRI sequences. Bony coverage of the semicircular canal in the coronal plane in NR-CT (**d**) and PETRA MRI sequence with inverted grayscale (**e**) and native grayscale (**f**): note the similarity between the STARVIBE sequence and NR-CT, even in the thinnest zone (white arrows). *CT* Computed tomography, *MRI* Magnetic resonance imaging, *NR* Normal resolution, *SHR* Super-high resolution. **Supplementary Fig. S3**. Sinodural angle imaged with different CT and MRI modalities (anatomic specimen) in sagittal plane. **a** SHR-CT showing the sinodural angle measurement method between the tegmen tympani superiorly and the sigmoid sinus inferiorly represented by two white lines. Sinodural angle imaged in NR-CT (**b**), STARVIBE (**c**), PETRA (**d**) and UTE (**e**) MRI sequences. CT, computed tomography; MRI, magnetic resonance imaging; NR, normal resolution; SHR, super-high resolution; UTE, ultrashort time of echo

## Data Availability

The datasets used and/or analyzed during the current study are available from the corresponding author upon reasonable request.

## Abbreviations

CI: Cochlear implantation
CTN: Chorda tympani nerve
MFN: Mastoid portion of the facial nerve
MRI: Magnetic resonance imaging
NR-CT: Normal-resolution computed tomography
PETRA: Pointwise-encoding time reduction with radial acquisition
RW: Round window
SHR-CT: Super-high-resolution computed tomography
SMF: Stylomastoid foramen
SSCC: Superior semicircular canal
STARVIBE: Radial volumetric interpolated breath-hold
UTE: Ultrashort time of echo
