# Supplementary material for: Comparison of CT-like MRI sequences for preoperative planning of cochlear implantation using super-high-resolution CT as a reference
Source: Eur Radiol Exp. 2025 Jan 2;9:1. doi: 10.1186/s41747-024-00538-x (PMC11695506; doi:10.1186/s41747-024-00538-x)
Supplement: Supplementary file 1 — Additional file 1: Supplementary Fig. S1. Right facial recess imaged with different CT and UTE-MRI sequences in the parasagittal plane (example in living patient). a SHR-CT showing the distance between the origin of the chorda tympani nerve (white dotted arrow) and the stylomastoid foramen measurement method. b NR-CT showing the chorda-facial angle measurement method between the mastoid portion of the facial nerve (MFN: white arrow) and the origin of the bony segment of the chorda tympani nerve (CTN: dotted white arrow). Facial recess composed of the MFN and the bony segment of the CTN in UTE sequences in inverted grayscale (c) and native grayscale (d). CT, computed tomography; MRI, magnetic resonance imaging; NR, normal resolution; SHR, super-high resolution; UTE, ultrashort time of echo. Supplementary Fig. S2. Superior semicircular canal and tympanic bony coverage in a 19-year-old patient followed for an operated cholesteatoma in NR-CT and STARVIBE MRI sequences (example in living patient). Superior semicircular canal bony coverage in the Pöschl plane in NR-CT (a) and PETRA reverse grayscale (b) and native grayscale (c) MRI sequences. Bony coverage of the semicircular canal in the coronal plane in NR-CT (d) and PETRA MRI sequence with inverted grayscale (e) and native grayscale (f): note the similarity between the STARVIBE sequence and NR-CT, even in the thinnest zone (white arrows). CT Computed tomography, MRI Magnetic resonance imaging, NR Normal resolution, SHR Super-high resolution. Supplementary Fig. S3. Sinodural angle imaged with different CT and MRI modalities (anatomic specimen) in sagittal plane. a SHR-CT showing the sinodural angle measurement method between the tegmen tympani superiorly and the sigmoid sinus inferiorly represented by two white lines. Sinodural angle imaged in NR-CT (b), STARVIBE (c), PETRA (d) and UTE (e) MRI sequences. CT, computed tomography; MRI, magnetic resonance imaging; NR, normal resolution; SHR, super-high resolution; UTE, ult [file 41747_2024_538_MOESM1_ESM.pdf]

Comparison of CT-like MRI sequences for preoperative planning of cochlear implantation using super-high resolution CT as a reference

ELECTRONIC SUPPLEMENTARY MATERIAL

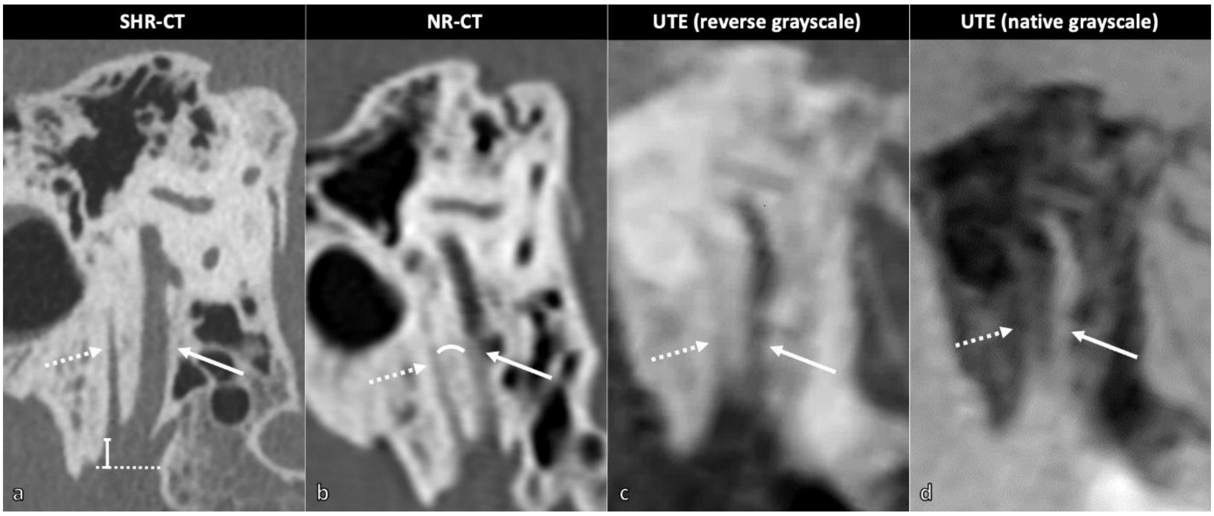

Supplementary Fig. S1. Right facial recess imaged with different CT and UTE MRI sequences in the parasagittal plane (example in living patient). (**a**) SHR-CT showing the distance between the origin of the chorda tympani nerve (white dotted arrow) and the stylomastoid foramen measurement method. (**b**) NR-CT showing the chorda-facial angle measurement method between the mastoid portion of the facial nerve (MFN: white arrow) and the origin of the bony segment of the chorda tympani nerve (CTN: dotted white arrow). Facial recess composed of the MFN and the bony segment of the CTN in UTE sequences in inverted grayscale (**c**) and native grayscale (**d**). *CT* Computed tomography, *MRI* Magnetic resonance imaging, *NR* Normal resolution, *SHR* Super-high resolution, *UTE* Ultrashort time of echo.

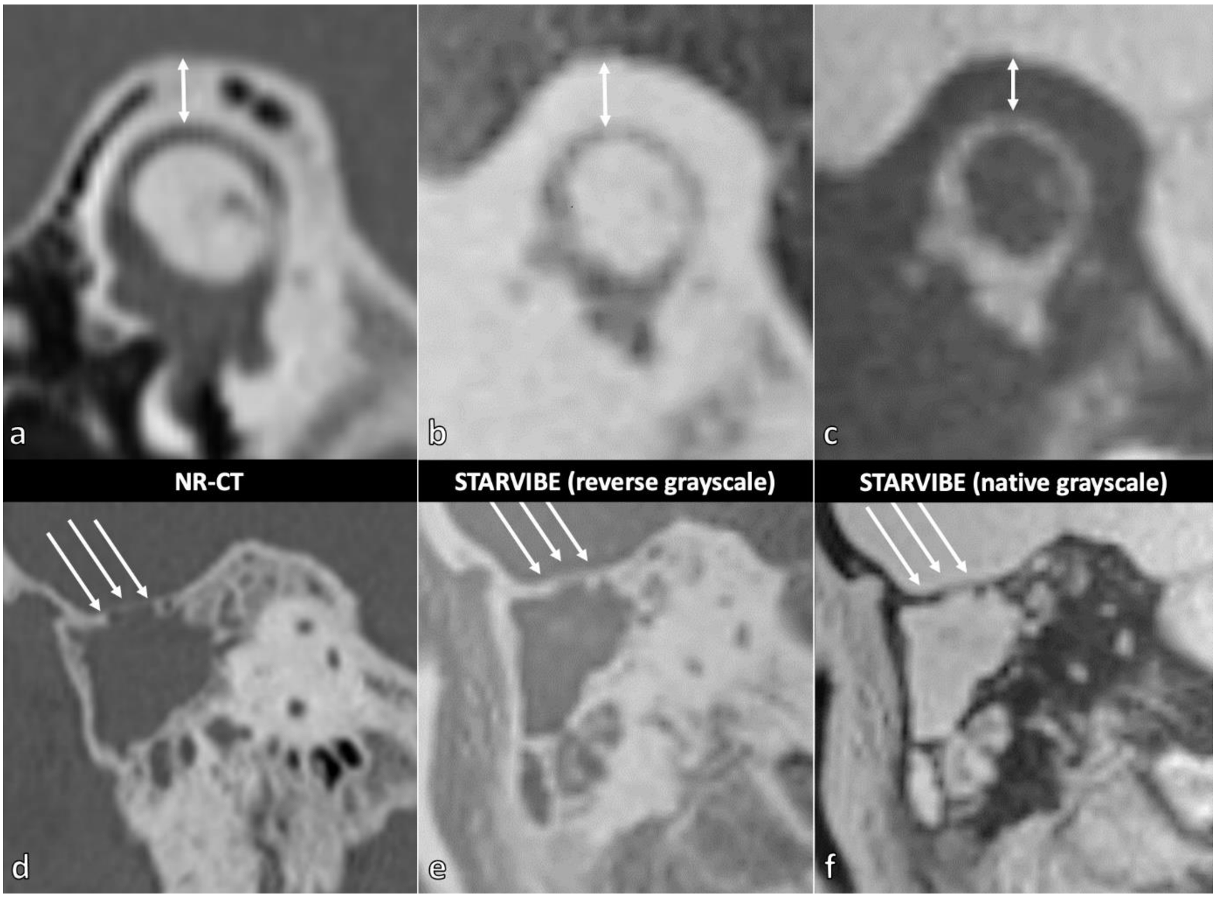

Supplementary Fig. S2. Superior semicircular canal and tympanic bony coverage in a 19-year-old patient followed for an operated cholesteatoma in NR-CT and STARVIBE MRI sequences (example in living patient).

Superior semicircular canal bony coverage in the Pöschl plane in NR-CT (**a**) and PETRA reverse grayscale (**b**) and native grayscale (**c**) MRI sequences. Bony coverage of the semicircular canal in the coronal plane in NR-CT (**d**) and PETRA MRI sequence with inverted grayscale (**e**) and native grayscale (**f**): note the similarity between the STARVIBE sequence and NR-CT, even in the thinnest zone (white arrows). *CT* Computed tomography, *MRI* Magnetic resonance imaging, *NR* Normal resolution, *SHR* Super-high resolution.

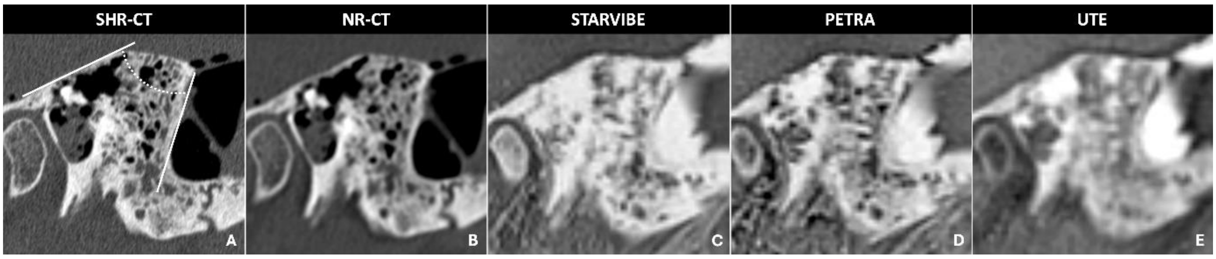

Supplementary Fig. S3. Sinodural angle imaged with different CT and MRI modalities (anatomic specimen) in sagittal plane. (**a**) SHR-CT showing the sinodural angle measurement method between the tegmen tympani superiorly and the sigmoid sinus inferiorly represented by two white lines. Sinodural angle imaged in NR-CT (**b**), STARVIBE (**c**), PETRA (**d**) and UTE (**e**) MRI sequences. *CT* Computed tomography, *MRI* Magnetic resonance imaging, *NR* Normal resolution, *SHR* Super-high resolution, *UTE* Ultrashort time of echo.
